# Strategies to improve engagement of ‘hard to reach’ older people in research on health promotion: a systematic review

**DOI:** 10.1186/s12889-017-4241-8

**Published:** 2017-04-21

**Authors:** Ann E. M. Liljas, Kate Walters, Ana Jovicic, Steve Iliffe, Jill Manthorpe, Claire Goodman, Kalpa Kharicha

**Affiliations:** 1 Research Department of Primary Care and Population Health, University College London, London, UK; 2 Social Care Workforce Research Unit, King’s College London, London, UK; 3 Centre for Research in Primary and Community Care (CRIPACC), University of Hertfordshire, Hatfield, UK

**Keywords:** Ageing, Health promotion, Inequalities, Older people, Oldest old, Ethnicity, Deprivation

## Abstract

**Background:**

This systematic review aimed to identify facilitators, barriers and strategies for engaging ‘hard to reach’ older people in research on health promotion; the oldest old (≥80 years), older people from black and minority ethnic groups (BME) and older people living in deprived areas.

**Methods:**

Eight databases were searched to identify eligible studies using quantitative, qualitative, and mixed research methods. Using elements of narrative synthesis, engagement strategies, and reported facilitators and barriers were identified, tabulated and analysed thematically for each of the three groups of older people.

**Results:**

Twenty-three studies (3 with oldest-old, 16 with BME older people, 2 within deprived areas, 1 with both oldest-old and BME, 1 with both BME and deprived areas) were included. Methods included 10 quantitative studies (of which 1 was an RCT), 12 qualitative studies and one mixed-methods study. Facilitators for engaging the oldest old included gaining family support and having flexible sessions. Facilitators for BME groups included building trust through known professionals/community leaders, targeting personal interests, and addressing ethnic and cultural characteristics. Among older people in deprived areas, facilitators for engagement included encouragement by peers and providing refreshments. Across all groups, barriers for engagement were deteriorating health, having other priorities and lack of transport/inaccessibility. Feeling too tired and lacking support from family members were additional barriers for the oldest old. Similarly, feeling too tired and too old to participate in research on health promotion were reported by BME groups. Barriers for BME groups included lack of motivation and self-confidence, and cultural and language differences. Barriers identified in deprived areas included use of written recruitment materials. Strategies to successfully engage with the oldest old included home visits and professionals securing consent if needed. Strategies to engage older people from BME groups included developing community connections and organising social group sessions. Strategies to engage with older people in deprived areas included flexibility in timing and location of interventions.

**Conclusions:**

This review identified facilitators, barriers and strategies for engaging ‘hard to reach’ older people in health promotion but research has been mainly descriptive and there was no high quality evidence on the effectiveness of different approaches.

## Background

Globally, the ageing population is growing, contributing to pressures on health and social care systems [1]. Health promotion interventions to assist older people in building and maintaining their physical and cognitive function can reduce the risks of disease and loss of independence [1]. Preventative strategies for chronic diseases associated with older age have therefore become a public health priority [2]. The World Health Organization (WHO), declares it a major challenge to promote health to all older people through health interventions targeting the diversity of health and functional states in older people, often driven by influences that are beyond the individual’s control such as genetic inheritance and physical and social environments [1]. The oldest old (people aged 80 years and over), older people living in deprived areas and older people from black and minority ethnic groups (BME) have more health problems and health care disparities compared to the general older population [3, 4]. The oldest old is the fastest growing age group in the population [5, 6], making them an important target for health interventions. Furthermore, this group is a diverse section of the population, ranging from relatively healthy, independently living individuals to very frail individuals with multiple diseases, poor physical functioning and cognitive problems, presenting unique challenges for undertaking research on health promotion [7], and thus they are often excluded from studies [8, 9]. However there is a growing body of evidence suggesting that the oldest old can gain substantially from various health interventions [10]. A systematic review has shown greater improvements from health promotion activities such as exercise in frail individuals aged 80–90 years compared to individuals aged 71–79 years suggesting that health interventions such as exercise classes can be beneficial to the oldest old [11]. Similarly, a meta-analysis has shown that balance training is effective in preventing falls in those aged 75 years and over [12]. However, no systematic reviews on the oldest old have to our knowledge considered a broad range of health promotion interventions within the review.

BME older people form a significantly increasing proportion of the ageing population in both Europe and North America [10, 13]. However, older BME groups have been underrepresented in clinical research [14, 15], and have reported greater difficulty accessing health and social care services [16, 17]. A recent meta-ethnographic analysis on barriers to physical activity among BME groups aged 18–65 years in the UK showed that physical activity was often seen as a formal separate activity and a part of Western culture external to their own lifestyle and difficult to incorporate into their lives. The authors suggested culturally sensitive health promotion interventions are crucial to increase physical activity levels in BME communities [18]. However we do not know if these findings apply in older people from BME groups. Furthermore, to our knowledge, no systematic reviews on older BME groups and engagement in broad health promotion interventions (that is, not limited to one intervention e.g. physical activity) have reviewed facilitators and barriers for engagement.

Multi-morbidity is more common and occurs 10–15 years earlier in older people who live in deprived areas compared to older people in affluent areas [19]. Nevertheless, health promotion interventions have not been employed extensively among older populations in deprived areas [8, 20], and to our knowledge no systematic reviews have reviewed engagement in research on health promotion in older people living in deprived areas.

Health inequity often underpins the diversity observed in older age and it has been suggested that disproportionate efforts should be made to reach sub-groups of older people that are particularly disadvantaged [1, 20]. The most effective engagement strategies and intervention features in reaching disadvantaged older people need to be systematically determined in order to target those who could benefit the most [21]. A systematic review therefore, focusing on facilitators, barriers and effective methods to engage the oldest old, older people from BME groups, and older people in deprived areas in health promotion provides a resource for current work and future research. This systematic review examines how researchers engage ‘hard to reach’ older people in research on health promotion. The findings provide insights on how ‘hard to reach’ older people could be engaged in health promotion interventions outside of the research context.

## Methods

### Search strategy and eligibility criteria

For this review, 8 electronic databases, MEDLINE, Cochrane Library, SCOPUS, EMBASE, PsychInfo, Social Sciences Citation Index (SSCI), Cumulative Index to Nursing and Allied Health Literature (CINAHL), and the Social Care Institute for Excellence’s Social Care Online (SCIE), were searched. The search strategy is outlined in Appendix A. Keywords were developed iteratively over several meetings with the research team involved in a wider study on engaging ‘hard to reach’ groups in health and well-being promotion, of which this review is one part. The team consisted of researchers with expertise in ageing and health and social care, a research assistant, a PhD student and two patient and public representatives who were involved at all stages of the wider study including seeking funding. Quantitative, qualitative and mixed-method studies of engagement of older people to research on health promotion published between 1 January 1990 and 31 December 2014 were included. In this paper, engagement refers to processes such as introducing, recruiting and retaining individuals into health promotion interventions. Systematic reviews and studies not exploring the topic of engagement in health promotion among at least one of the three sub-groups defined (oldest old aged 80 years and over, BME older people, older people in deprived areas) were excluded. Studies were also excluded if: the mean age of participants was under 50 years; the age of participants was not specified (studies referring to the participants as ‘older people’ were included); participants were dwelling exclusively in nursing/residential care homes; or if the study focused on selected populations with a specific medical condition (e.g. dementia) or learning disabilities/intellectual disabilities. Searches were restricted to studies in English published between January 1990 and December 2014. References retrieved through the systematic searches were managed using Endnote X7 reference manager software.

### Study selection

**Fig. 1:**
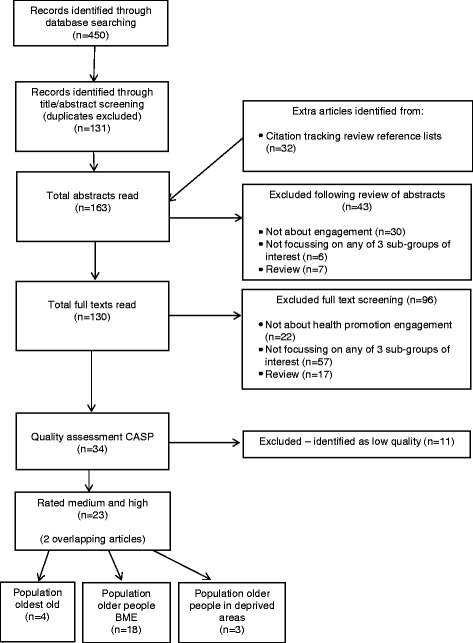
Study selection flowchart

The study selection procedure is outlined in the PRISMA diagram, Fig. 1 (‘Study selection flowchart’). Following de-duplication of studies obtained from the database searches, 163 abstracts were independently screened for eligibility by two researchers (AJ and AL). Any disagreements were resolved by a third researcher (KK). Abstracts not meeting the inclusion criteria were excluded, resulting in 130 studies which were read in full text. Studies not meeting the criteria were excluded, leaving 34 studies for quality assessment using the Critical Appraisal Skills Programme (CASP) guidelines [22], which enabled the studies to be classified into high, medium or low quality. Eleven studies were classified as low quality and removed because, for example, the authors referred to facilitators and barriers in previous studies but did not report or discuss potential facilitators and barriers derived from data from their own study or it was unclear whether the potential facilitators and barriers mentioned were reported by the participants or hypothesised by the researchers. This resulted in a total of 23 studies for analysis (16 studies rated as medium and 7 studies rated as high). Studies covering more than one of the three ‘hard to reach’ groups were analysed for the groups targeted and facilitators and barriers for each specific group were analysed separately.

### Data extraction and analysis

Narrative methods using thematic analysis can be used to synthesise quantitative and qualitative studies to identify and bring together the main, recurrent or most important issues or themes arising from the literature [23]. For this study, data extraction and analysis were guided by principles of narrative synthesis, including tabulation and thematic analysis [24]. The data were extracted inductively using a standardised data extraction form. Two researchers independently identified facilitators and barriers and grouped them into themes for each sub-group of older people (the oldest old, BME groups and those living in deprived neighbourhoods/from lower socio-economic backgrounds). The themes were identified by the same two researchers and then discussed, further developed and agreed with the team. A narrative approach was used to allow handling of a wide range of evidence from quantitative and qualitative research divided into facilitators and barriers.

## Results

This review reports potential facilitators, barriers and strategies to engagement in research on health promotion in groups of older people known to participate less, including the oldest old, older people from black and minority ethnic groups (BME), and older people living in deprived areas. From this data we have extracted key potential strategies, reported as having been successfully employed by authors to increase engagement with health promotion across the three groups. However findings should be interpreted with caution as only one study included in this review has formally tested the effectiveness of these approaches in an experimental study.

### Description of the studies identified

Twenty-three articles were included in the systematic review (Tables 1 and 2). Three studies were of the oldest old, 16 studies were of BME older people and 2 studies were of older people in deprived areas. Two studies overlapped; one including both the oldest old and BME older people, and one including both BME older people and older people in deprived areas. Ten studies were of quantitative methods (e.g. descriptive studies, survey design), 12 qualitative studies (e.g. focus groups) and 1 mixed-methods study. Themes about older people’s engagement in health promotion were categorised as facilitators, barriers and strategies for engagement and are described below for each group. Facilitating factors and barriers were defined as factors that may or may not be modifiable but that impact on engagement; strategies are defined as methods or approaches deliberately employed as part of studies to increase engagement/recruitment into research on health promotion. A summary of themes of potential facilitators, barriers and strategies for engagement for each of the three ‘hard to reach’ groups are presented in Table 3.

**Table 1:** Characteristics of studies on the oldest old and older people living in deprived areas

Reference number	Author(s)	Country	Study design	Participant population group (oldest old, BME, deprived area)and age	Potential facilitators (themes)	Potential barriers (themes)
[4]	Davies, K. et al. 2010	England	Quantitative, descriptive study on recruitment methods	Oldest old, ≥85 years	Family involvement, trust and respect, recruitment and maintenance strategy, location, flexible assessment, participant consent strategy	Mortality risk, poor health, unwillingness, interfering family members
[25]	Dyall, L. et al. 2013	New Zealand	Quantitative, structured questionnaires, descriptive study on recruitment methods	Oldest oldand BME (Māori), ≥85 years	Family involvement, trust and respect, recruitment and maintenance strategy	Mortality risk, poor health, unwillingness
[26]	Ewart, C.V. et al. 2001	United States	Quantitative, descriptive study on recruitment methods	Oldest old, 65–105 years	Trust and respect, recruitment and maintenance strategy, flexible assessment	Mortality risk, poor health, unwillingness, interfering family members, poor location
[5]	Pascucci, M. et al. 2012	United States	Quantitative, descriptive study and structured survey	Oldest old, 80–101 years	None	Poor health, poor location
[44]	Buijs, R. et al. 2003	Canada	Qualitative, focus groups and individual interviews	Deprived area, 61–90 years	Motivation, adaptable service	Poor health, lack of interest
[40]	Martinez, I.L. et al. 2009	United States	Qualitative, focus groups	Deprived area and BME (African American), 61–89 years	Motivation, free food	Poor health, inaccessibility, costs
[21]	Mills, K.M. et al. 1996	United States	Quantitative, descriptive study and structured interviews	Deprived area, ≥62 years	Introductory meeting at the housing estate	Poor health, lack of interest, letter invitation

**Table 2:** Characteristics of studies on older people from black and minority ethnic groups (BME)

Number	Author(s)	Country	Study design	Participant population group (oldest old, BME, deprived area)and age	Potential facilitators	Potential barriers
[28]	Arean, P. A. et al. 2003	United States	Quantitative, quasi-experimental comparative study	BME(African American, Latino) ≥65 years	Ethnical and cultural aspects, community connections, recruitment and maintenance strategy	Poor recruitment and engagement strategy
[42]	Bynum, S. A. et al. 2012	United States	Quantitative, structured interviews	BME(African American, Hispanic) ≥50 years	None	Lack of confidence, lack of knowledge
[30]	Carlson, M. et al. 2014	United States	Quantitative, descriptive study embedded in RCT	BME(African American, Hispanic) 60–95 years	Ethnical and cultural aspects, community connections, social support, providing transportation	Other priorities
[27]	Coleman, E.A. et al. 1997	United States	Qualitative, descriptive study on recruitment methods	BME (African American) ‘older people’ - age not specified	Community connections, incentives, recruitment and maintenance strategy	Poor recruitment and engagement strategy
[25]	Dyall, L. et al. 2013	New Zealand	Quantitative, structured quesionnaires, descriptive study on recruitment methods	Oldest oldand BME (Māori), ≥85 years	Trust, ethnical and cultural aspects, benefits to participant’s family, community connections, recruitment and maintenance strategy	Poor health, other priorities, poor recruitment and engagement strategy
[32]	Ellish, N. J. et al. 2009	United States	Quantitative, descriptive study on recruitment methods	BME(African American) ≥65 years	Community connections, familiar location	Poor recruitment and engagement strategy
[38]	Holland, C.A. et al. 2008	England	Mixed methods, questionnaire survey and telephone interviews	BME (Indian, African-Caribbean) ≥50 years	Incentives, social support, familiar location	Poor health, poor recruitment and engagement strategy, lack of transportation
[37]	Horne, M. et al. 2013	England	Qualitative, focus groups	BME (Indian, Pakistani) 60–70 years	Social support	Poor health, lack of transportation, feeling too old, lack of motivation, cultural and language barriers, lack of confidence
[29]	MacEntee, M.I. et al. 2002	United States	Qualitative, descriptive study on recruitment methods	BME(Vietnamese, Spanish, Cantonese-speaking, Punjabi-speaking) 60–75 years	Community connections, recruitment and maintenance strategy	Cultural and language barriers
[41]	Manthorpe, J. et al. 2009	England	Qualitative, focus groups	BME (Asian, Black, Mixed, Other) ‘older people’ - age not specified	None	Cultural and language barriers
[40]	Martinez, I.L. et al. 2009	United States	Qualitative, focus groups	Deprived area and BME (African American), 61–89 years	None	Lack of transportation, too tired, costs
[36]	Mathews, A. et al. 2010	United States	Qualitative, focus groups	BME (African Americans, American Indians,Latinos, Chinese, Vietnamese) 50–90 years	Benefits to the individual, social support	Poor health, other priorities, lack of transportation, costs, feeling too old, lack of confidence, lack of knowledge
[39]	Prohaska, T.R. et al. 2000	United States	Qualitative, structured interviews	BME (African American) ≥55 years	Social support	Other priorities, lack of motivation
[31]	Stineman, M.G. et al. 2010	United States	Quantitative, randomised controlled trial	BME (African American) ≥65 years	Community connections, social support, providing transportation	Poor health
[33]	Sullivan-Marx, E.M. et al. 2011	United States	Qualitative, descriptive study and structured questionnaires	BME (African American) ‘older people’ - age not specified	Trust, benefits to participant’s family, recruitment and maintenance strategy, providing transportation	Poor health, other priorities, lack of motivation
[34]	Walcott-McQuigg, J.A. & Prohaska, T.R. 2001	United States	Qualitative, focus groups	BME (African American) ≥55 years	Benefits to the individual, incentives, social support, providing transportation	Other priorities, lack of motivation
[35]	Wilcox, S. et al. 2005	United States	Qualitative, focus groups	BME (African American) 67.5 +/−9.2 years	Benefits to the individual	Lack of transportation, too tired, costs, feeling too old, lack of motivation, lack of confidence, lack of knowledge
[43]	Williams, M.P. 1996	United States	Qualitative, focus groups and individual interviews	BME (African American) ≥55 years	Incentives, familiar location, recruitment and maintenance strategy	None

**Table 3:** Summary of themes of potential facilitators, barriers and strategies for engagement for the oldest old, older people from BME groups and older people living in deprived areas

Oldest old		
*Potential facilitators*	*Potential barriers*	*Strategies for engagement*
• family involvement (e.g. engaging with family carers) • flexible assessment (length and time of sessions) • trust	• poor health • tiredness • unwillingness to engage in research on health promotion • lack of motivation • lack of support from family members • inaccessibility (lack of transport to research site, lack of facilities for in-home sessions)	• recruitment via primary care by known and trusted professionals • respectful and empathic approach • shorter visits over several months • ongoing face-to-face and written contact • home visits • check participants status with their GP
Older people from BME groups		
*Potential facilitators*	*Potential barriers*	*Strategies for engagement*
• cultural and ethnic aspects e.g. connections to the targeted community and matching participants and researchers by ethnicity • trust • personal benefits and benefits to participant’s family • social support from family, friends, staff and peers	• having other priorities • lack of transportation • costs • poor health • lack of motivation • cultural and language barriers • lack of confidence • lack of knowledge	• familiar location • word-of-mouth • information easy-to-read (bullet point format, photo of research team • introductory meetings • providing transportation • monetary incentives • friendly competitions
Older people in deprived areas		
*Potential facilitators*	*Potential barriers*	*Strategies for engagement*
• encouragement by others • personal interest in participating • complimentary refreshments	• poor health • inaccessibility • costs • lack of interest	• offering adaptable approach according to participants’ needs • social relationships between participants and researchers to create comfortable environment • face-to-face contact

### Facilitators for engaging the oldest old

Family involvement in the form of the research team engaging with family carers was demonstrated to be important when undertaking research with the oldest old [4]. This included providing opportunities for family members to discuss benefits and risks of their older relative’s participation [25]. Further, allowing for flexibility in timing and length of the intervention was found to facilitate health assessments in participants reporting fatigue [4, 26]. One study reported that 90-min sessions were initially offered but some participants benefited from having several shorter sessions [4]. The risk of fatigue further resulted in researchers conducting the most relevant part of the assessment first [4].

### Barriers for engaging the oldest old

On an individual level, poor health was a barrier reported in all studies [4, 5, 25, 26]. Similarly, feeling too tired stopped people from taking part [4, 5] or resulted in participants only undertaking a shorter version of the assessment [4]. Family members did not always seem to share subjects’ commitment to participate in research [26]. In addition, family members may also ignore the request or be slow in providing participation assent for a relative who lacks decision making capacity, which may require more time and resources [4]. In-home sessions included barriers such as lack of facilities for example, a firm surface to lie down on for certain health and research measurements [26].

### Strategies for engaging the oldest old

Recruitment via primary care health professionals who are known and trusted by participants was successful [25, 26]. Invitation letters sent from the university undertaking the research study to potential participants asking for a structured interview, physical examination and access to medical records generated a good response rate for some recruitment sites [25]. Recruitment materials from the research team with photographs of the researchers accompanied with a letter from the local primary care clinic conveyed trustworthiness and encouraged prospective participants to contact the research team directly, rather than the primary care clinic, which also reduced workload [4]. Respectful and empathic telephone calls by researchers approaching prospective subjects were reported as a successful recruitment strategy if a home visit had been undertaken before or after the phone call [4, 26]. Carrying out the recruitment process over several stages over several months may also have facilitated participation by minimising subject burden and limiting resource intensity for the research team [26]. It was reported that ongoing face-to-face and written communication aided long-term engagement, and the research team also found it useful to ask the participant to nominate someone with whom the research team could liaise in case the participant would lose capacity during the course of the study [4]. In one study, location of data collection was discussed, and the oldest old stated a preference for home visits over external venues such as hospital or other clinical settings [4].

Most studies on the oldest old unsurprisingly reported problems with high mortality risks [4, 25, 26], including identifying potential participants who died before being contacted for recruitment [4, 25], and mistakenly having tried to enrol participants who had recently died [26]. Strategies reported as useful in minimising these risks included checking their status with their GP and posting recruitment letters within 24 h after such checks [4]. It was further reported that the research team needs to know how to communicate with family members of potential study participants who died very soon after recruitment letters had been posted to minimise distress to the family [4].

### Facilitators for engaging older people from BME groups

Several studies reported that developing strong connections to the targeted community and its leaders was essential as it resulted in greater acceptance of the study and the study team, increasing engagement [25, 27–32]. Matching participants and researchers by ethnicity facilitated communication and allowed for mutual understanding of cultural practices [25, 28, 30], and this resulted in a greater number of individuals being willing to take part compared to non-ethnically matched recruiters [28]. Trust included initial contact made by known and trusted primary care health professionals [25], and reassurance from clinicians that sickness or disability would not be a problem when engaging in, for example, physical activity interventions [33].

Health promotion interventions that participants thought would personally benefit their health [34, 35] as well as their families [25, 33] including sessions targeting mental and physical health (e.g. weight loss and back problems) [36], were valued by participants. Social support from family, friends and healthcare professionals was important in encouraging participants to enrol as well as remain in the study [34, 37]. Social support also included group-based health interventions [30, 31, 36, 38, 39], which were preferred to individual sessions because of their social element [30, 31].

### Barriers for engaging older people from BME groups

Many studies reported that having other priorities such as family responsibilities including caring for grandchildren (particularly women) [34, 36, 39], schedule or timetabling conflicts [39] or lack of time [25, 30, 33, 39] prevented participation. Some older people from BME groups were reluctant to receive home visits [38]. Barriers to engage in health promotion further included poor health being too burdensome for subjects to participate [25, 31, 33, 35–38, 40], and feeling too old to benefit from health promotion [35–37]. In one study participants thought the health assessment was too long and asked for a partial assessment [25]. Cultural and language barriers included the contact person of the research team speaking English only [29], having difficulty obtaining information due to lack of translated materials [41] or a lack of large print translated versions of publicity material [41]. In addition, religious practices including fasting [37, 41] and mixed-sex sessions prevented participation in physical activity interventions [37]. Lack of confidence mainly referred to physical activity interventions and included lack of belief in their own physical ability [37], poor balance [37], and risk of injuries / fear of falling [35–37]. Lack of confidence furthermore included fear of embarrassment of taking part in a specific screening test [42]. Lack of knowledge referred to participants who perceived risks of participating in the screening tests [42], but also referred to less intrusive interventions such as physical activity due to not being aware of the benefits of leading an active lifestyle [36] and choosing not to participate in physical activities for fear of ‘overdoing it’ [35, 36].

### Strategies for engaging older people from BME groups

Participants were more likely to take part if the research on health promotion was held in a familiar place [43]. Recruitment in churches [43] and senior centres [32, 38] resulted in the highest enrolment rates. In contrast several studies reported problems recruiting potential participants when using leaflets [27] and local radio and newspapers publicity [28, 32, 38]. Face-to-face and gatekeeper referrals were the most successful recruitment methods in one study [28]. Recruitment was also more likely to be successful if participants had heard about the study by word-of-mouth first [27, 43]. In one study invitation letters sent from the research university generated greater response rates for some but not all recruitment sites [25], whereas another study reported that pictorial, “easy-to-read” leaflets with information in a bullet point format and photos of the research team were the most successful print media [43]. Introductory meetings about the study at community centres in combination with posters and newspaper advertising facilitated recruitment [29, 32] and encouraged prospective participants to telephone the research team to enrol [29]. This strategy was reported as more effective than ‘cold calls’ [29]. However, another study reported that telephoning potential participants was more effective than distributing leaflets, letters, organising presentations and TV adverts [27]. Also, integrating health promotion activities into an existing health care programme and coordinating participants’ schedules facilitated recruitment and engagement/attendance [33]. Several studies reported that providing transport to the research/intervention site facilitated engagement [30, 31, 33, 34]. In two studies, monetary incentives such as vouchers to show appreciation for participation were reported as very useful [27, 43] Two other studies found that providing prizes as part of friendly competitions embedded into the physical activity interventions encouraged participation [34, 38].

### Facilitators for engaging older people in deprived areas

Factors that facilitated prospective participants to enrol and attend included being encouraged by peers to take part and receiving positive feedback from those already engaged [44]. Another facilitator was having a personal interest in participating in specific health interventions which they thought could benefit them personally and included sessions about avoiding loneliness or learning new things [40]. Older men in deprived areas reported that free food motivated them to attend [40].

### Barriers for recruiting and engaging older people in deprived areas

A barrier reported in all studies targeting older people in deprived areas was deteriorating health due to chronic diseases limiting their mobility [40, 44], and sensory impairments causing communication problems [21]. Inaccessibility referred to inadequate access and public transport, especially for those with mobility problems, preventing participation [40]. Older people in deprived areas further reported not being interested in attending activities associated with a cost. [40] Lack of interest was another barrier which included having other priorities [44], forgetting to attend sessions [44], and finding no value in participation [21].

### Strategies for engaging older people in deprived areas

An adaptable approach that allowed for participants’ needs, which changed over time, including offering home visits if desired, was reported as a particularly important strategy for engaging older people in deprived areas [44]. Adaptability also included addressing transport barriers which contributed to positive social relationships between staff and participants [44]. Such positive social relationships further created a comfortable environment which was seen as important and valued by participants and staff [44]. One study reported that invitation by letter was a less effective strategy compared to telephone recruitment. [21] In contrast, face-to-face recruitment could be effective; holding an introductory meeting at the participants’ housing estate where the study and staff were presented to prospective participants resulted in two thirds of those attending the meeting being enrolled to the programme [21].

## Discussion

A wide range of facilitators, barriers and strategies for engagement in research on health promotion by the oldest old, older people from BME groups and those living in deprived areas have been identified from this review.

### Key themes shared across groups

Three key themes were shared across the three sub-groups; poor health, face-to-face contact and family/peer influence on participation. Firstly, all three ‘hard to reach’ groups reported poor or deteriorating health as a barrier to participation including studies reporting participants feeling too old to benefit from health promotion [35–37]. Secondly, whilst findings on effective recruitment methods were somewhat different between the three sub-groups and even included inconsistent findings within older people from BME groups, face-to-face contact was consistently reported as a successful engagement strategy for all three sub-groups [4, 21, 28], and should be considered when targeting any of these three sub-groups. Thirdly, studies on all three sub-groups reported that participants’ decision on whether to engage in health promotion was often influenced by family (oldest old, older people from BME groups) and/or peers (older people from BME groups, older people in deprived areas). This finding stresses the importance of linking research on health promotion for older people into their community, connecting with other relevant local organisations, and establishing a good relationship with participants’ families.

### Key differences between the groups

Key differences between the three ‘hard to reach’ groups included location/agency for recruitment, access, and individual versus group sessions. Our findings showed that the oldest old were successfully recruited into research on health promotion through primary care [4, 26], older people from BME groups through religious organisations, senior centres and community leaders [32, 38, 43], and older people in deprived areas through meetings organised in their residential area [21]. These findings suggest that different locations for recruitment need to be considered for each of these sub-groups in order to reach them. Further, providing transport to the research site was a successful strategy to overcome inaccessibility as well as financial barriers that applied to both older people from BME groups [31, 33], and older people in deprived areas [44]. Rather than being offered transport to an external site, the oldest old preferred research staff to visit them in their homes [4]. Home visits were also reported to be beneficial to overcome participation barriers among older people in deprived areas [44]. In contrast, older people from BME groups preferred group sessions for social reasons [30, 31]. The preferences of the three groups may well overlap which suggest that a flexibility of approach may be warranted.

It is noteworthy that many of these findings may also apply to the general older population. For instance, offering an adaptable service and allowing for flexibility in time and length of contact are likely to be attractive to all older people, as is involving people already known to and trusted by prospective participants. Similar findings have previously been reported in an earlier systematic review targeting the general older population concluding that mental health interventions need to be tailored to the individual’s abilities and preferences [45].

To our knowledge no previous systematic reviews on the oldest old, older people from BME groups and older people living in deprived areas have reviewed engagement in a broad range of health promotion interventions. The findings of this systematic review add to the current literature on ‘hard to reach’ older age groups in several ways. First, in respect of the oldest old, this systematic review has generated a more detailed understanding of the views of the oldest old by identifying both facilitators and barriers to engagement, than has previously been described for this group. For example, our review shows that involving family members may facilitate engagement in health promotion by encouraging the older person to take part. However it also reveals that family involvement may act as a barrier if family members do not share the older person’s commitment to participate in health promotion initiatives [26]. This builds on previous systematic reviews on for example participation in physical activity and falls prevention interventions reporting supportive family involvement alone [46, 47]. Our approach which did not restrict literature to certain health promotion interventions such as physical activity or falls prevention may have allowed for a wider range of facilitators and barriers to be considered, plausibly providing a more comprehensive picture of the role of family involvement. Second, personal benefits of participating in health promotion were a facilitator reported by both older people from BME groups and older people living in deprived areas. This supports previous research on the general older population showing that benefits believed to improve the individual’s personal health strongly motivate engagement in exercise classes [48]. Finally, our systematic review has showed that different activities were perceived as beneficial by older people from BME groups compared with older people in deprived areas; older people from BME groups thought they would personally benefit from activities such as losing weight and recovering from back pain [36], whereas older people in deprived areas believed they would personally benefit from sessions about avoiding loneliness or learning new things [40]. This finding suggests that the focus of health promotion interventions should be designed specifically to the interests of individual ‘hard to reach’ groups of older adults.

### Strengths and limitations

This study’s strengths include targeting three different sub-groups of older people known to participate less in health promotion. This is to our knowledge one of the first systematic reviews identifying facilitators and barriers for engagement in health promotion not restricted to a specific health promotion activity such as exercise in the oldest old, older people from BME groups and older people living in deprived areas, often identified as ‘hard to reach’.

Limitations to this study include that older people from BME groups were not classified into different BME subgroups depending on ethnicity, making it difficult to apply our findings to specific BME groups, and did not take into account migrant status. Most of the BME studies included refer to Black African Americans. Differences in patterns of migration, ethnic composition, settlement and healthcare systems in North America compared with Europe or elsewhere have not been discussed. Also, many BME groups live in deprived areas [49, 50], suggesting that the two sub-groups BME and deprived areas may overlap. Not all studies had a primary aim of systematically identifying facilitators and barriers for the success of the recruitment and engagement into research on health promotion, and data from some studies [36, 37] were therefore sparse. The number of studies on the oldest old and those living in deprived areas was small making it difficult to generalise and compare findings across the three groups of older people. Finally, this review focussed on three specific ‘hard to reach’ groups in older age but we acknowledge that other sections of the older population may be under-represented in health promotion interventions and research, for example people with dementia [51] and older people with learning disabilities/intellectual impairment [52].

## Conclusions

This systematic review has identified numerous facilitators and barriers for recruiting and engaging three sub-groups of the older population; the oldest old, older people from BME groups and older people living in deprived areas, to research on health promotion. Key themes shared across all three sub-groups included poor health, face-to-face contact and family/peer influence on participation. Key differences between the groups were location/agency for recruitment, access and individual vs group sessions. In addition, we found specific facilitators and barriers for each particular sub-group. The findings of this systematic review are of importance to consider in practice to maximise engagement of these three ‘hard to reach’ groups into research on health promotion. We suggest that researchers report on the specific strategies that they find useful or otherwise to enlarge the evidence base on this subject. More studies are particularly needed of health promotion for the oldest old and older people living in deprived areas and future research should also investigate potential differences between older people from different BME groups.

## Appendix

**Table 4:** Search terms used

Key concept	Search terms	MeSH terms
Population	old age aging/ageing old* adult* old* people elder* (inc. elder, elders, elderly) geriatric* senior* pensioner* over 65*/over sixty five*/over sixty-five* 65+ veteran*	Aged Aging Age – over 80
Health promotion	health promotion* behavio* chang* healthy aging/ageing health education lifestyle wellbeing health campaign* health prevent* health protect* primary prevent*	Health promotion Behavioural changes
Engagement	engag* participat* involv* partak* recruit* retention retain enrol*	Patient participation Patient selection Research subject recruitment
‘Hard to reach’ groups	ethnic* (inc. ethnic, ethnicity) BME minority depriv* over 85*/over eighty five*/over eighty-five* 85+ socioeconomic	Ethnic groups Minority groups Age – over 80 Social class Poverty areas Socioeconomic factors

## Abbreviations

BME: Black and minority ethnic
CASP: Critical appraisal skills programme
CINAHL: Cumulative index to nursing and allied health literature
EMBASE: Excerpta Medica dataBASE
MEDLINE: Medical literature analysis and retrieval system online
PRISMA: Preferred reporting items for systematic reviews and meta-analyses
SCIE: Social care institute for Excellence’s Social Care Online
SSCI: Social Sciences Citation Index
UK: United Kingdom
WHO: World Health Organization
